# Natural and synthetic fiber reinforced recycled aggregate concrete subjected to standard fire temperature

**DOI:** 10.1016/j.heliyon.2024.e39676

**Published:** 2024-10-22

**Authors:** Balamurali Kanagaraj, Shinu Shaji, Meshach Jafrin, Samuvel Raj R, N. Anand, Eva Lubloy

**Affiliations:** a Department of Civil Engineering, Karunya Institute of Technology and Sciences, Coimbatore, India; b Department of Construction Materials and Technologies, Faculty of Civil Engineering, Budapest University of Technology and Economics, Budapest, 1521, Hungary

**Keywords:** Concrete, Recycled aggregates, Fiber, Porosity, Strength, Elevated temperature

## Abstract

The increasing scarcity of natural materials and rising construction costs have prompted the need for alternative materials that can match the performance of conventional aggregates. This study explores the use of recycled aggregate concrete (RAC) as a sustainable alternative, supplemented with natural and synthetic fibers to overcome the inherent strength limitations caused by the residual cement paste on the aggregate surface. To enhance the mechanical and thermal stability of RAC, steel, polypropylene, and coconut fibers were incorporated. The rheological, hardened, and post-fire properties of fiber-reinforced RAC were thoroughly investigated. After 28 days, the porosity of RAC with fiber reinforcement was observed to be less than 2 %. The concrete was also exposed to elevated temperatures as per ISO 834 guidelines to assess its thermal performance. Key parameters such as mass loss, crack width, porosity, and strength degradation were analyzed. Image analysis was used to track surface modifications and measure surface porosity after heating. Among the fiber types, steel fiber reinforced concrete exhibited superior mechanical and thermal performance, followed by coconut fiber reinforced concrete, making coconut fibers a viable natural alternative to synthetic fibers. RAC without fibers displayed the highest porosity (20 % and 32 %) after exposure to 821 °C and 1029 °C, respectively. Strength degradation ranged from 40 to 50 % after heating to 821 °C, increasing to 74–80 % at 1029 °C, irrespective of fiber type. This research highlights the potential of fiber-reinforced RAC for structural applications, offering a sustainable solution with comparable strength and durability to conventional concrete.

## 1 Introduction

Due to its advantages, concrete was employed as a construction material around the globe, with its overall use was estimated to be ten billion tons on an average per year, which consume around eight billion tons of filler materials, for its production process [1]. On the other side, increase in the infrastructural activities and demolition of age-old structures (i.e. service life completed structures) leads to the production of demolition wastes [2]. This scenario can be seen most frequently in developing nations. Due to lack of awareness regarding the waste management, these wastes are often dumped in open lands and disturbing the rainwater runoff, thereby indirectly affecting the surrounding eco-system [3]. It is essential to recycle the waste and should be employed as source materials for activities like basement filling materials; aggregates for concrete, which results in preserving the eco-system and promoting conservation of natural resources [4].

Recycled aggregate Concrete (RAC) produced by crushing the existing concrete debris, comprises of adherence of cement mortar on its surface, which has the tendency to absorb higher water during the manufacturing process of aggregates [5]. Higher water absorption characteristics of this RAC during the concrete mixing process, results in increasing the water demand, beyond the mix proportion calculations [6]. Adding extra water in the concrete mix tends to bleeding and segregation, thereby decline the strength of the mix when compared to the mix comprised with virgin materials [7]. Further, the adhered mortar on the aggregate surface will have interfacial transition zone (ITZ), when these RAC was employed for the concrete production process, a new ITZ will be developed [8]. It is known that, during loading the failure of the concrete was originated from the ITZ, so concrete developed with RAC will have two ITZ's; upon loading the weaker ITZ tends to collapse first, thereby initiating the failure of the concrete as soon as possible [9]. This might be one of the possible reasons for the lower strength attainment for the concrete developed with RAC [10].

The behavior of concrete under fire scenario is one of the crucial studies; which encompasses the performance of concrete under severe environment [11]. Due to increasing fire accidents in buildings, fire studies are important and necessary guidelines has to be proposed to safeguard the structure during fire accidents [12]. Fire accidents not only involved in the loss of human life's, but it involves huge amount of money invested for building the structure. Structural fire is not a frequent incident, and degree of flammability depends on the combustible materials present inside the structure; therefore, the temperature during the fire accidents is unpredictable [13]. Studies are undertaken to examine the behavior of concrete at various temperatures from 100 to 1000 °C [14]. Since concrete is a poor conductor of heat and electricity, it has the tendency to retail its strength up to 400 °C, and further it can withstand its physical structure at 1000 °C but loosing majority of its strength [15]. Behavior of concrete under elevated temperature is still debatable because of the rate of heating involved during the fire studies.

It is known fact that concrete attains its strength by heat of hydration reaction process; which is accelerated by water curing for a period of 28-d. Because of which some portion of water molecules are being stored in the pores/voids inside the concrete matrix [16]. When subjecting the concrete with high pore water to elevated temperature, the water molecules tend to vaporizes and initiate the hydration reaction process with the unreacted cementitious materials; which is commonly called as autoclave curing process [17]. The strength development of concrete will be restricted to certain temperature, beyond which the calcium silicate hydrate (CSH) gel tends to decompose and leading to strength loss in the concrete system [18]. Due to lower binder proportion in the normal strength concrete, the concrete matrix developed will have the tendency of pore/void formation. Whereas these types of concrete subjected to elevated temperature possess interlinking of voids/pore in the matrix system; which paves a path for the escape of pore water pressure during heating [19]. In the case of high dense concrete's, the presence of pores/voids inside the concrete matrix will be low; because of these characteristics, the strength and other long-term performance of the concrete will be superior, compared to the traditional type of concrete [11]. When these types of concrete subjected to fire exposure, the vapors developed inside was unable to get out of the concrete matrix; accumulation of this pressures inside the pore, exceeds the tensile strength of the concrete thereby resulting in bursting. It is often called as spalling of concrete during fire tests.

Further, incorporation of fibers in the concrete system tends to enhance the tensile strength of the concrete, and this type of concrete often called as fiber reinforced concrete (FRC). And it acts as bridging between crack, developed during placing, setting, and loading of concrete [20]. Therefore, to prevent the spalling of concrete during fire scenario, fibers were introduced in the concrete system [21]. Moreover, fibers are classified into two main categories, namely, natural and synthetic fibers. Synthetic fibers are often costlier than natural fibers. Fibers like carbon and aramid are quite costlier; because of the cost associated during the manufacturing process. Further the type, aspect ratio, and the proportion of the fiber plays a major role in the strength improvement in the concrete system [22]. When compared to all the fiber types, carbon and aramid fibers possess superior strength, contrastingly incorporation of such fibers in the concrete tends to increase the production cost of the concrete, and results in uneconomical construction practices [23]. Fibers like polypropylene fibers, steel fibers, are belonging to synthetic category, but these fibers are not costlier as that of carbon fibers; therefore, these fibers were currently employed in the construction sector to enhance the harden and long-term performance of the concrete system [24].

Wang et al. [25] examined the strength softening behavior and energy dissipation capacity of recycled aggregate concrete with the incorporation of steel and polypropylene fibers. The experimental study shows that, the strength softening of the specimens were increased by 30 % for steel fiber blended mix and 25 % for polypropylene fiber blended mix. Moreover, the incorporation of steel fibers enhances the internal morphology and mechanical characteristics of the recycled aggregate concrete blended mix [26]. In addition to the above, Wang et al. [27] investigated the impact loading behavior of fiber reinforced recycled aggregate concrete, based on the experimental test, it was found that the impact behavior of fiber reinforced recycled aggregate concrete was superior than the conventional concrete. Incorporation of micro steel fiber enhances the harden properties of the matrix, showing that the steel based fibrous materials has the positive potential to enhance the mechanical characteristics of concrete [28].

### 1.1 Research significance

In the present study, virgin coarse aggregate was replaced with RAC to examine the performance of the harden characteristics of the concrete. In addition, fibrous materials were introduced into the concrete to examine the performance. A comparative examination of natural fiber (coconut fiber) and synthetic fibers (steel and polypropylene fiber) were made. The main significance of the paper, aims to understand the fire behavior of fiber reinforced RAC blended concrete (FRAC) and to compare its performance with the conventional. The concrete specimens were heated as per ISO 834 guidelines, four heating temperatures were adopted namely, 821 °C,925°C,986°Cand1029°C..

Further, the research evaluates the mass loss, porosity, crack width, and residual mechanical properties of both RAC and natural aggregate concrete after exposure to high temperatures, providing a detailed comparison between different fiber-reinforced concrete mixes under elevated temperature conditions. Quantitative models are developed to predict the relationship between porosity and mass loss for RAC and natural aggregate concrete, with and without fiber reinforcement. These models offer a predictive tool for fire-related damage in concrete, which is beneficial for practical applications in fire-resistant construction design. The study's findings suggest that fiber-reinforced RAC is a viable material for use in fire-prone environments, with steel fibers providing the best overall performance. Coconut fibers are also identified as a promising sustainable and cost-effective alternative for structural applications where fire resistance is crucial.

## 2 Methodology

**Fig. 1:**
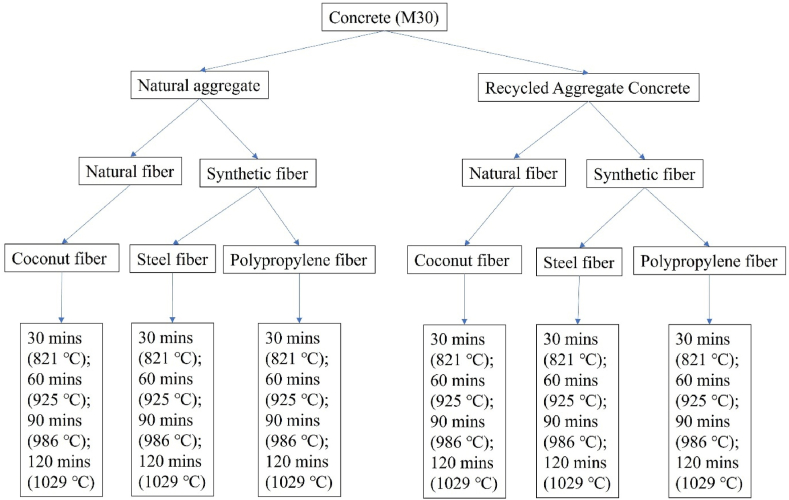
Methodology adopted in the present study.

The methodology adopted in the present investigation is shown in Fig. 1; which showcase the type of fiber incorporation, type of aggregate employed for the production as well as examining the rheological and harden characteristics of concrete. In addition, information regarding heating the concrete specimens at various durations and intensities were shown. In the present study, the dosage of fiber (steel, polypropylene and coconut) was fixed constant as 2 % for all the concrete mixes. Based on the literature source, 2 % dose of fibers was found to be optimal, therefore, in the present study the same was employed to examine the harden properties of concrete before and after subjecting to elevated temperature [20,[29], [30], [31], [32], [33], [34]].

## 3 Materials

**Fig. 2:**
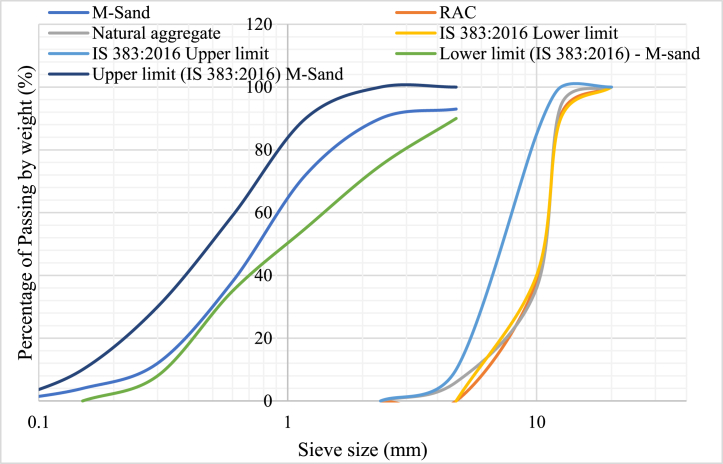
Particle size distribution of natural and recycled aggregates.

Portland cement (PPC) was used as binder medium for the production of concrete, in the present study. Due to scarcity of river sand, manufactured sand commonly called as M-sand of size <4.75 mm were used as fine aggregates, in accordance with IS 383 [35]. The particle size distribution of the natural and RAC was plotted in Fig. 2. Crushed quarry stones of size <20 mm were employed as coarse aggregate. The RAC obtained through the laboratory demolished cubes specimens of size <20 mm was also used as filler material in the present study. The properties of the natural aggregates and RAC was reported in Table 1. Natural fiber (coconut fiber) and synthetic fiber (steel fiber and polypropylene fiber) were employed for the production of concrete. The aspect ratio of steel, polypropylene and coconut fiber was 50, 315 and 125.

**Table 1:** Properties of aggregates.

Aggregate type	Size (mm)	Water Absorption (%)	Specific gravity
M-Sand	4.75	0.71	2.6
Natural coarse aggregate	10–20	0.5	2.79
RAC	10–20	0.65	2.81

## 4 Methods

### 4.1 Mix design

Eight types of concrete mixes were developed with natural aggregate and RAC. The control specimens (C-N) developed using natural aggregate, with a design strength of 30 MPa (M30). M30 refers to the concrete mix having the cube compressive strength of 30 MPa. The proportion of RAC involved in the development of concrete was 100 %. C-N and C-R represents concrete with natural and recycled aggregate. The mix proportions involved in the present study was illustrated in Table 2. It is known fact that, RAC will have the tendency to absorb more water, thereby making water deficiency for lubricating the mix. Therefore, to mitigate this issue, the RAC materials were presoaked in water for 1 h and dried in room temperature for 1 h; after drying, the RAC were employed in the concrete manufacturing process. C-N-S, C-N-P, and C-N-C represents concrete with natural aggregate and steel, polypropylene, and coconut fiber.

**Table 2:** Mix proportion for RAC blended concrete.

Materials	C-N	C-R	C-N-C	C-N-S	C-N-P	C-R-C	C-R-S	C-R-P
Cement (kg/m^3^)	400	400	400	400	400	400	400	400
Fine Aggregate (kg/m^3^)	820	820	820	820	820	820	820	820
Natural Coarse aggregate (kg/m^3^)	1060	–	1060	1060	1060	–	–	–
Recycled Coarse aggregate (kg/m^3^)	–	1060	–	–	–	1060	1060	1060
Coconut fiber (kg/m^3^)	–	–	8	–	–	8	–	–
Steel fiber (kg/m^3^)	–	–	–	8	–	–	8	–
Polypropylene fiber (kg/m^3^)	–	–	–	–	8	–	–	8

### 4.2 Rheology test

To examine the rheological characteristics of the concrete, after mixing the concrete in the mixer machine, the concrete samples were tested using slump cone apparatus, as per ASTM C 143 [36]. The test was carried by pouring the concrete in the slump cone, layer-by-layer then the cone was lifted vertically, to measure the height difference between the cone and the concrete, which represents the slump value.

### 4.3 Hardened test

After finding the workability of the concrete, the fresh mix was transferred to the cubical molds of size 150 mm × 150 mm as per IS 516 [37]. The concrete was filled layer-by-layer, to ensure proper filling in the cube mold. Then the concrete filled mold was transferred to the table vibrator, to ensure proper compacting was achieved. After 24 h of setting, the concrete was demolded from the molds, and allowed to cure in the curing tank for 28-d to initiate the heat of hydration, which is essential for the strength development of the mix.

#### 4.3.1 Density

The samples were taken from the curing tank after 28-d of curing, and allowed to dry in room temperature for 24 h, for surface drying [38]. The weight of the concrete samples was measured using a weigh balance. And the density of the developed concrete was estimated using Equation (1).

(1) Density = (mass/volume)

#### 4.3.2 Sorptivity

Sorptivity also called as rate of water absorption, which represents the rate of water ingression in the concrete specimens with respect to time, in line with ASTM C 1585 [39]. 100 mm diameter disc specimens with a height of 50 mm were employed to measure the sorptivity. The specimen was coated with a layer of bitumen around the side surface, followed by thin plastic sheet wrapping, to ensure the water penetration in the concrete specimen, only through capillary action (from bottom to top approach). Then the specimens were placed in water, and the rate of absorption was measured at various interval time. Sorptivity was estimated using Equation (2). Where I is the absorption rate; mt is the mass of the specimen with respect to time; a is the specimen area; and d is the water density.

(2) I = m_t_/(a/d)

#### 4.3.3 Porosity

Pore formation inside the concrete is unavoidable, due to various factors and responsible for strength degradation of concrete. The porosity of the concrete was examined using Equation (3), proposed by Shen and Xu [40]. Concrete samples were dried in the oven for a temperature of 100 ± 5 °C to achieve a constant mass, to measure the porosity level.

(3) $$P=\frac{\left(m_{w}-m_{d}\right)}{\rho v}$$

Where, p is the porosity of the mix; m_w_ is the saturated mass; m_d_ is the dried mass; v is the volume; ρ is the water density.

#### 4.3.4 Compressive strength

Compression test was performed in the cubical specimen of size 150 mm × 150 mm, the specimens after the curing period, were loaded in the compression testing machine, as per ASTM C 39 [41]. The load was applied vertically, allowing the cube to crush. Compressive strength was estimated by finding the ratio between failure load of the specimen to the cross-sectional area.

#### 4.3.5 Fire test

**Fig. 3:**
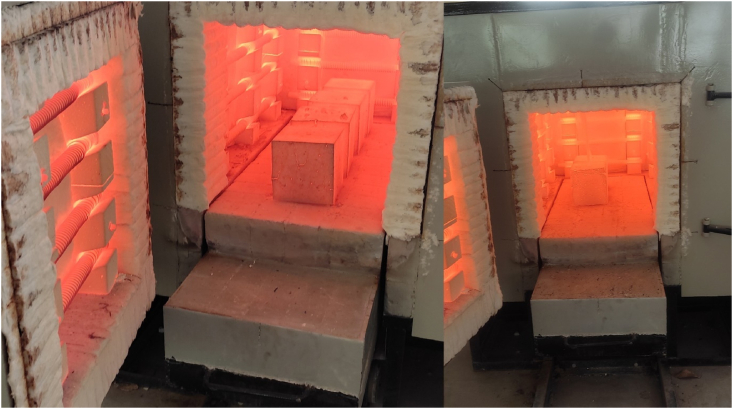
Heating of concrete specimens in the furnace.

The samples were exposed to elevated temperature as per ISO 834 guidelines. As per the code, ISO 834 [42], four different duration were adopted to study the behavior of concrete, namely, 30, 60, 90, and 120 min. An electric furnace of 1200 °C capacity, was used to heat the specimens, as shown in Fig. 3. For each temperature the duration was pre-determined by ISO 834 guidelines, for example exposing to 821 °C requires 30 min of duration; for 925 °C requires 60 min; for 986 °C requires 90 min and for 1029 °C requires 120 min; based on which the concrete samples were heated in the computerized electric furnace in the present investigation.

## 5 Results and discussion

### 5.1 Fresh properties

As stated above, the rheology of the freshly developed concrete was evaluated using slum cone apparatus. Where, the newly developed concrete mixture was placed in layers and compacted to maintain the uniformity, then the cone was lifted vertically up and the behavior of concrete after the cone removal was visually seen. The variation in the height between the cone and concrete was examined for all the concrete types. The slump value of C-N samples was seen to be 80 mm, whereas for the sample made with RAC i.e. C-R shows a slump value of 85 mm; the increase in the slump value of the C-R sample may be attributed due to pre-soaking effect. The slump of C-N-S, C-N-P, and C-N-C was found to be in the range between 60 mm and 75 mm. In the case of concrete blended with RAC, the slump value of C-R-S, C-R-P, C-R-C was found to be marginal in the range of 70–75 mm, respectively.

### 5.2 Density

**Fig. 4:**
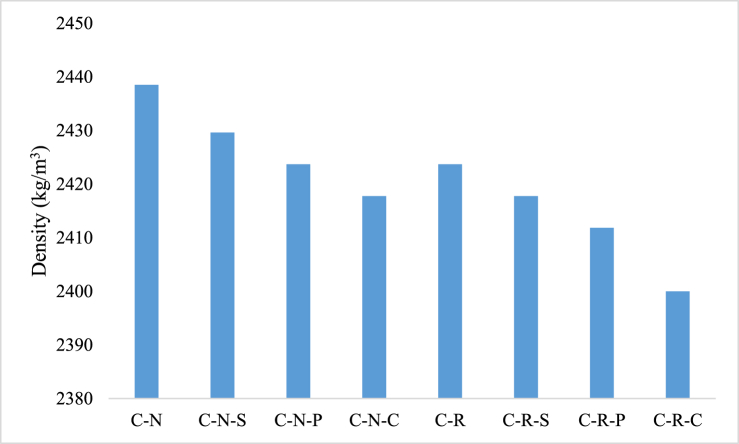
Density of concrete blended with natural aggregate and concrete with RAC (with and without fibers).

After 28-d of curing, the density of the developed concrete was examined for all the concrete specimens. The concrete made with natural aggregate (C-N) possess a density of 2438 kg/m^3^. The density of the developed concrete shows minor variations; which could be attributed varying the proportions of coarse aggregates in the concrete during manufacturing process. Concrete samples were mixed in the laboratory available mixer machine, to maintain the uniformity in mixing process. While transferring the freshly prepared concrete from the mixer machine to the molds; the proportion of the filler materials might be altered, due to which the minor fluctuations in the density of the concrete was observed. In the case of C-R mix, the density of the concrete was 2423 kg/m^3^. The density of the concrete mixes with natural aggregate and RAC (with and without incorporation of fibers) were illustrated in Fig. 4. Whereas the fiber incorporated mixes i.e. C-N-S, C-N-P, and C-N-C mix possess a density in the range between 2417 and 2429 kg/m^3^. In addition to the concrete with conventional aggregates, the density of the mix with RAC and fibers were 2400 and 2417 kg/m^3^.

### 5.3 Sorptivity

**Fig. 5:**
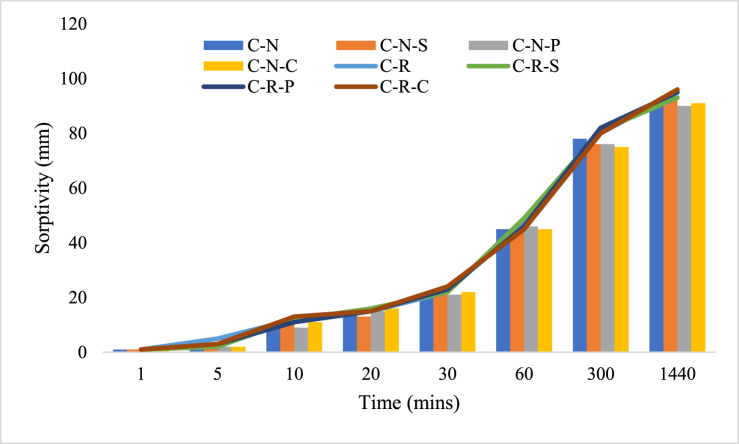
Sorptivity of concrete samples blended with natural aggregate and RAC.

Sorptivity measure the rate of water absorption through capillary action with respect to time, which is one of the important factors which has to be considered when a structure is constructed in high water table area. The concrete specimens after 28-d of curing were employed for examining the rate of water absorption. The concrete specimen is prepared in such a way that the water penetration inside the concrete was only through the capillary action and not through the sideward direction. Concrete blended with natural as well as RAC materials and the fiber incorporated mix were subjected to sorptivity exposure. The natural aggregate blended mix shows quite lower sorptivity value when compared to RAC blended mix. The variation amongst the natural and RAC blended mix was found to be about 5 %, further the fiber incorporated mix possess slight fluctuations; this could be due to the varied proportion of the matrix in the specimen. The sorptivity values of the concrete specimens with natural aggregate and RAC (with and without the incorporation of fibers) were plotted in Fig. 5.

### 5.4 Porosity

**Fig. 6:**
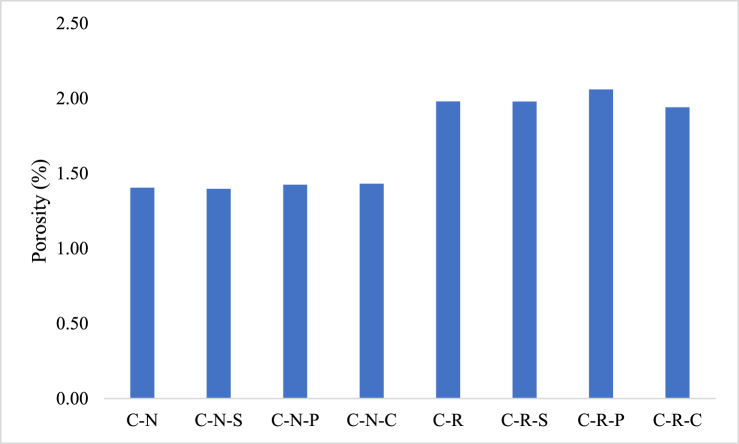
Porosity of the concrete with natural & synthetic aggregates and fibers.

Porosity plays a significant role in the strength development of the concrete mix [43]. During the concrete production process addition of water, lower than the required proportion leads to very stiff mix; which makes the concrete difficult to transfer and place [44]. Moreover, compacting the concrete of very stiff mix, leads to formation of honeycombs and pores inside the concrete, responsible for poor homogeneity thereby making the concrete weak [45]. Therefore, it is essential to develop a dense mix free from voids. Generally, in the case of normal strength concrete the elimination of pores inside the matrix is difficult, but concrete can be developed with minimal pores; this could be possible, by adding suitable admixtures, and proper mix proportioning [46]. In the present investigation, concrete developed with natural aggregates (C-N mix) possess a porosity of 1.4 %, and for the concrete mix developed with RAC (C-R mix) shows a porosity level of 1.98 %. The C-R mix possess 29 % higher porosity when compared to the C-N mix; this could be attributed by the presence of old mortar on the top of the aggregate. The porosity range of both the concrete types was found to be minimal, showing <2 % and this could be the reason for the strength attainment of the RAC blended concrete. The porosity values of the concrete blended with natural and RAC was illustrated in Fig. 6.

The porosity of the C-N-S specimens was found to be 1.4 %, resembling porosity of C-N mix. The C-N-P mix shows a porosity of 1.42 %, which is 1.39 % higher than the C-N mix and the porosity of the C-N-C mix was found to be 1.43 %, showing 1.89 % higher the porosity of the C-N and C-N-S mix. The variation could be attributed by varied proportion concrete mix ingredients. In the case of fibers incorporated RAC blended concrete i.e. C-R-S mix shows a porosity of 1.98 %, similar to the concrete with the natural aggregate, the RAC blended steel reinforced concrete resembles the porosity of the C-R mix. The porosity of the C-R-P mix was found to be 2.06 %, which was 3.93 % higher than the C-R mix. 1.94 % porosity was observed for the C-R-C mix; which was 1.94 % lower than the C-R mix and C-R-S mix. In comparing the porosity of the concrete with natural and RAC, C-R-S possess 29.3 % higher porosity than C-N-S mix. In the case of C-R-P and C-R-C mix: C-N-P mix possess 31 % lower porosity and C-N-C mix possess 26 % lower porosity. Moreover, all the concrete types possess lower porosity level; therefore, the strength of the concrete will not be affected upon ageing and subjecting to harsh environments.

### 5.5 Compressive strength

The strength of the concrete was evaluated in two curing periods; 7- and 28-days. The C-N specimens possess a 7-d strength of 17.2 MPa; whereas C-R specimens possess a strength of 13.7 MPa, resulting 20.3 % lower strength compared to the conventional concrete. After 28-d of curing, the strength of the C-N specimens was 32.6 MPa, and for C-R specimens the strength was 29.6 MPa. The 28-d strength of RAC blended concrete possess 9 % lower strength than the conventional aggregate. The lower strength of the RAC blended specimens was attributed by dual ITZ. As stated above, RAC possess layer of old mortar at its surface, comprising an ITZ; when this type of material was used for the production of concrete, due to the adhesive behavior of cement paste, a new layer of ITZ will be formed at the top of the old mortar. When these types of concrete subjected to loading after the curing period, the weaker ITZ tends to collapse and leading to lose in bond between the binder and filler medium.

In the case of FRC specimens, the strength of C-N-S specimens was found to be 20.5 MPa after 7-d of water curing; for C-R-S specimens, the strength was 14.62 MPa, showing a lower strength of 28.6 %. For C-N-P specimens, the strength was 33.89 MPa and 31.8 MPa for C-R-P specimens. In the case of coconut fiber reinforced samples, the strength was 32.91 MPa and 31.05 MPa. The polypropylene and coconut fiber blended specimens possess similar strength with marginal variation. In comparison between natural and RAC blended FRC specimens, the difference in strength was 14 % for C-N-S and C-R-S. C-N-P specimens possess 6.16 % superior strength than C-R-P specimens; meanwhile C-N-C specimens possess 5.65 % higher strength compared to C-R-C specimens. This shows that natural materials possess superior performance than the recycled once. But incorporation of fibers into the RAC blended concrete (C-R-S) resembles strength as that of the conventional concrete (C-N). C-R-P and C-R-C attains the target strength of the C-N mix with 2.4–4.7 % variation. This shows that the RAC can be used for the construction of structures with the addition of fibers; the type and proportion of fibers had an influence in attaining the concrete of structural grade.

### 5.6 Post fire behavior

#### 5.6.1 Surface modification

After subjecting to elevated temperature, the concrete specimens were allowed to cool through air-cooling process. Change in surface of the concrete was attributed due to the temperature variation between inner concrete core and the specimen surface. It is known fact that, concrete is not a good conductor of heat and electricity, therefore, the heat inside the concrete core will quite lower than the heat at the surface [47]. The variation in heat, tends to develop thermal gradient inside the concrete, as a result these thermal gradients are responsible for the crack formation on the surface. In the present case, the C-N specimens possess lower surface crack, when compared to the C-R specimens. There is no positive sign of spalling in all the concrete specimens. There is no sign of change in color of the concrete specimens; i.e. all the concrete specimens possess similar color before and after subjecting to heating. The concrete specimens i.e. C-N and C-R specimens after exposing to 821 °C, shows grey color, and moreover, after subjecting to 1029 °C, the color of the specimen resembles the same.

#### 5.6.2 Crack width

**Fig. 7:**
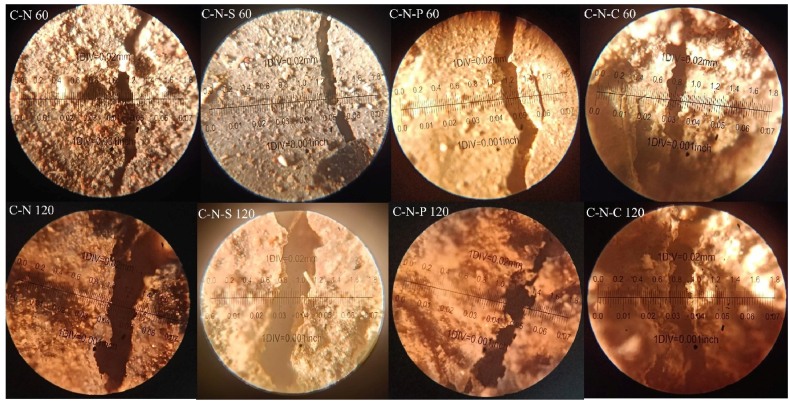
Natural aggregate blended concrete with and without incorporation of fibers.

The first step in examining the fire behavior is through visualizing the crack pattern and width developed at the concrete surface. It is a general fact, that concrete with higher crack width will be termed as severely damaged concrete, and based on the crack width, it can be viewed that the concrete's residual strength. Higher the crack width, lower the residual strength; contrastingly lower the crack width higher would be the residual strength. At 821 °C, the C-N specimen shows minor crack width, whereas C-R specimens possess higher crack width. The higher crack width of the C-R specimens was attributed by the presence of higher moisture content inside the concrete matrix; higher moister of the C-R matrix was due to the pre-soaking effect of the RAC specimens before concrete production. Increased moisture content in the RAC specimens, upon heating tends to increase the pore pressure inside the concrete, and thereby resulting higher crack formation at the concrete surface. Similar behavior of the C-N and C-R specimens were seen after exposing to 1029 °C. The crack of natural aggregate blended concrete after exposing to various temperature exposures were plotted in Fig. 7.

**Fig. 8:**
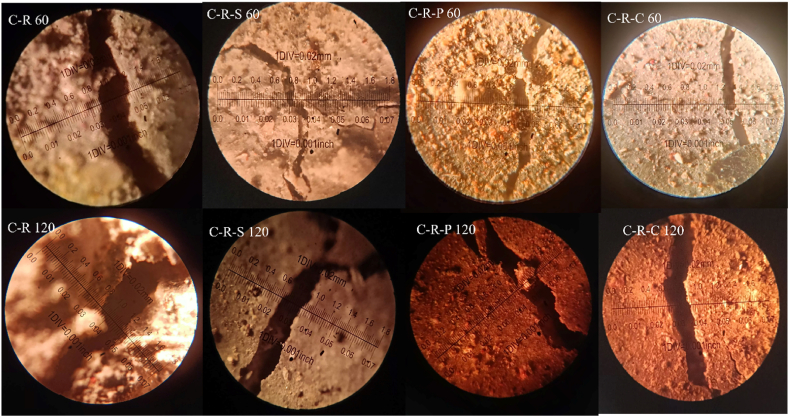
RAC blended concrete with and without incorporation of fibers.

**Fig. 9:**
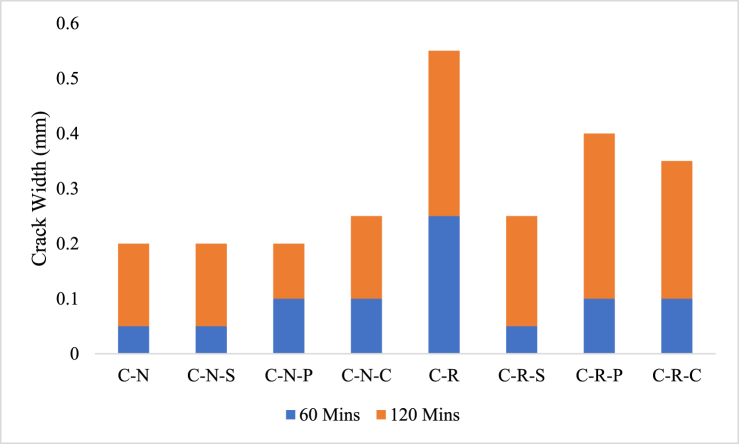
Crack width of concrete with and without incorporation of fibers.

After exposing to heating, the natural aggregate blended concrete i.e. C-N mix shows a crack width of 0.05 mm at 821 °C and 0.15 mm at 1029 °C. Further in the case of C-N-S mix, the crack width was seen to be 0.05 mm and 0.15 mm at 821 °C and 1029 °C, showing the steel fiber reinforced concrete resembles similar to that of the concrete without fibers. The C-N-P and C-N-C mix shows a crack of 0.1 mm after 821 °C, whereas the polypropylene fiber reinforced concrete shows lower crack width at 1029 °C, when compared to coconut fiber reinforced concrete. For the concrete blended with RAC (C-R mix), shows a crack width of 0.25 mm and 0.3 mm after subjected to 821 °C and 1029 °C. Fig. 8, depicts the concrete blended with RAC. The C-R-S mix shows a crack width of 0.05 mm at 821 °C and 0.2 mm at 1029 °C; meanwhile, the C-R-P and C-R-C mix shows a crack width of 0.1 mm after exposing to 821 °C, further increase in the intensity of heating increases the crack width of about 0.3 mm and 0.25 mm after 1029 °C of heating, as plotted in Fig. 9.

In the case of FRC specimens, i.e. C-N-S, C-N-P and C-N-C specimens, the crack formation was limited; due to the bridging effect of the fiber. At 821 °C, hairline cracks were seen on the specimen surface, whereas increasing the temperature tends to increase the width of the surface cracks for C-N-P and C-N-C specimens, for C-N-S specimens the width of the crack was quite lower than that of C-N-P and C-N-C specimens. In the case of RAC blended fiber reinforced mixes, the polypropylene mix and the mix without fiber resembles similar crack width after subjecting to 1029 °C. In general, steel fibers have high melting point when compared to polypropylene fiber. At a temperature of 1029 °C, the polypropylene fiber may have the tendency to melt, and results in reducing the bridging effect of fibers.

#### 5.6.3 Mass loss

**Fig. 10:**
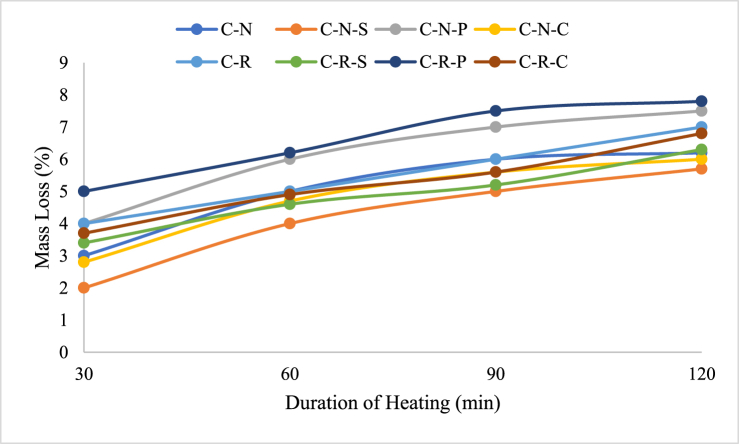
Loss in mass of concrete specimens with and without fibers after temperature exposure.

The mass loss of concrete with natural aggregate and RAC was depicted in Fig. 10. It can be seen in the figure that the concrete blended with natural aggregate possess lower loss when compared to the concrete blended with RAC. The loss of C-N mix after exposing to 821 °C was 3 %, whereas for the mix exposed to 1029 °C was 6.2 %, this shows that the percentage increase was doubled after 120 min. In the case of C-R specimens, the loss in mass was 4 % at 821 °C and 7 % for the specimens exposed to 1029 °C. In comparison with natural aggregate and RAC; RAC blended concrete possess 25 % higher loss after exposing to 821 °C. And after exposing to a temperature of 1029 °C, the loss percentage for RAC blended mix was 11.4 % higher than conventional aggregate based concrete.

For C-N-S specimens, the loss was 2 % at 821 °C and 5.7 % for 1029 °C exposed specimens, which shows a drop in mass loss when compared to the C-N specimens of about 8.06 %. The C-N-P specimens possess an increase in loss of 4 % at 821 °C and 7.5 % at 1029 °C; this shows that the polypropylene fibers blended specimens show 17.3 % higher loss when compared to C-N specimens. The C-N-C specimen shows a loss of 2.8 % at 821 °C, and 6 % at 1029 °C, denoting 3.2 % lower loss compared to C-N based specimens. Whereas, C-R-S mix possess a loss of 3.4 % at 821 °C and 6.3 % after exposing to 1029 °C. C-R-P and C-R-C mix, the loss was 5 % and 3.7 % after exposing to 821 °C; after subjecting to 1029 °C, the loss was 7.8 % and 6.8 %, respectively. In comparison with the fiber reinforced C-N and C-R specimens the loss was found to be in the range between 3.8 % and 11.7 %. C-R-S specimen possess 9.5 % higher loss than C-N-S specimens; C-R-P specimens possess 3.8 % higher loss than its counterpart; meanwhile C-R-C possess 11.76 % loss than C-N-C specimens.

The loss in mass of the concrete after subjecting to elevated temperature is mainly attributed by the evaporation of water molecules present inside the matrix [48]. Generally, concrete requires water for mixing of all the concrete ingredients and to increase the easiness while doing work. Apart from that, cement-based concrete requires water for heat of hydration, which is responsible for the strength development of the mix [49]. The water which binds the chemical reaction, and additional water remains in the pores after curing will be subjected to loss when the concrete is subjected to elevated temperature exposure [50]. The loss in mass was associated due to RAC based materials, which requires higher amount of water when compared to the conventional aggregate; this could be attributed by the adherence of cement paste at the surface of the aggregate [48].

#### 5.6.4 Residual strength

**Fig. 11:**
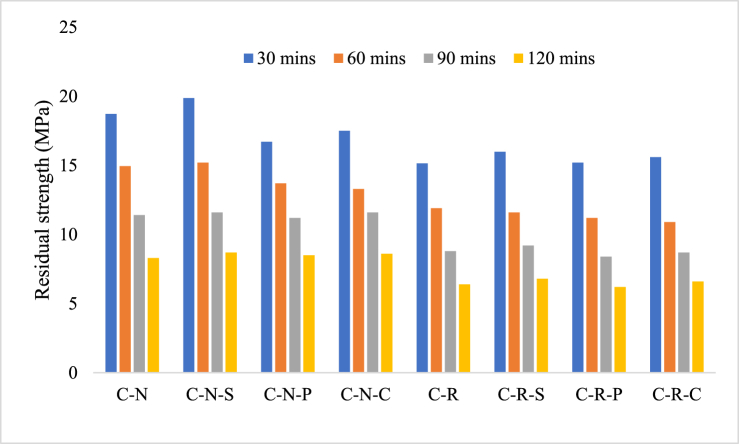
Residual strength of concrete after subjecting to temperature exposure.

As stated earlier, the crack development on the concrete surface was due to the effect of thermal gradient; this gradient was developed inside the concrete through thermal incompatibility between the concrete core and the surface. The residual strength of C-N mix after subjecting to 821 °C was found to be 18.72 MPa, showing 42.5 % loss when compared to unheated specimens. For the specimen exposed to 1029 °C, the residual strength was 8.3 MPa, showing a loss of 74.5 %. In comparison between 821 °C and 1029 °C exposed specimens, the loss variation was found to be 55.6 %. Representing, increase in the heating temperature increase the loss from 821 °C to 1029 °C. The residual strength of RAC blended mixes i.e. C-R specimen possesses a residual strength of 15.15 MPa after 821 °C of heating, with a loss of 49 % compared to unheated specimen. Meanwhile increasing the temperature to 1029 °C, the residual strength was recorded as 6.4 MPa, showing 78.4 % loss when compared to unheated specimens. 58 % loss was attributed by the RAC blended specimens when temperature increased from 821 °C to 1029 °C. The residuals strength of the concrete after heating was illustrated in Fig. 11.

The residual strength of fiber incorporated concrete specimens, i.e. C-N-S specimens shows 19.87 MPa after 821 °C, denoting 43 % losses compared to unheated specimens. 8.7 MPa was observed for the specimen exposed to 1029 °C, with a loss of 77 % to that of unheated specimens. The strength loss comparison between 821 °C and 1029 °C was 56 %. Meanwhile, the strength of C-N-P specimen was found to be 16.7 MPa, representing 51 % loss. At 1029 °C, the residual strength was 8.5 MPa, and the loss associated with the unheated specimen was found to be 75 %. 49 % loss was noted when the heating temperature was increased from 821 °C to 1029 °C. In the case of C-N-C specimens, the residual strength was found to be 17.5 MPa, after 821 °C of heating with a loss of 47 % compared to unheated specimens. Whereas for 1029 °C the exposed specimens possess a residual strength of 8.6 MPa, representing 74 % loss. Increasing the temperature from 821 °C to 1029 °C, results in 51 % loss in strength.

**Fig. 12:**
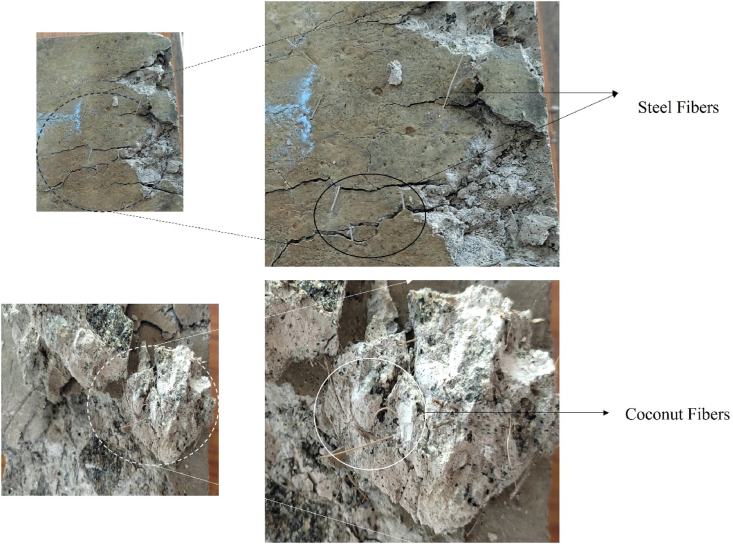
Presence of fiber inside the concrete after subjecting to temperature exposure.

In the case of RAC blended mixes i.e. the residual strength of C-R-S specimen after 821 °C was 15.9 MPa (50.2 % loss was seen after heating). After exposing to 1029 °C, the residual strength was 6.8 MPa (with a loss of 79 %); approximately 57 % of loss was associated when the concrete was heated from 821 °C to 1029 °C. C-R-P specimens possess 15.2 MPa after 821 °C of heating (with a loss of 52 %) and 6.2 MPa for the specimens subjected to 1029 °C (with a loss of 81 %). Meanwhile, the residual strength of C-R-C specimens was found to be 15.6 MPa after 821 °C (with a loss of 50 % when compared to unheated specimens) and 6.6 MPa for the specimens exposed to 1029 °C (with a loss of 79 %). In addition, the percentage loss in strength when the temperature increased from 821 °C to 1029 °C was found to be 59 % for C-R-P specimens and 58 % for C-R-C specimens. The presence of fibers namely, steel and coconut fibers is visible in the concrete after subjecting to 1029 °C, whereas the concrete blended with polypropylene fiber, after demolition there is no sign of fiber presence. This shows that the coconut fiber reinforced fiber has the higher potential in maintaining the stability of concrete similar to steel after subjected to elevated temperature exposure. The fiber present inside the concrete was viewed in Fig. 12.

The loss in strength of the concrete was mainly due to the decomposition of the CSH gel, which is responsible for the strength development of the concrete during hydration reaction process. At a temperature of 821 °C, most of the free pore water and chemically bound water tends to evaporates, causing a distress inside the concrete; the distress developed inside the concrete tends to reduce the bond between the binder and the filler medium [51]. Degradation of bond between the binder and the filler is one of the factors responsible for strength loss of the concrete. The de-bonding effect was associated with the breaking of ITZ inside the matrix and up on loading, the fractured ITZ tends to break the structure completely during initial period of loading [52]. Further increase in the temperature tends to fasten the above process.

#### 5.6.5 Porosity

**Fig. 13:**
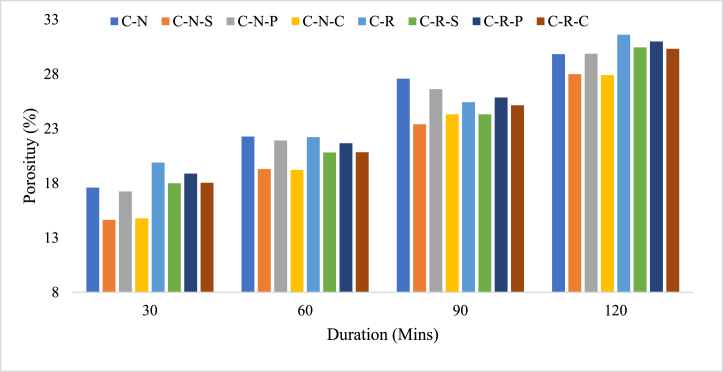
Porosity range of concrete specimens after heating.

The strength of the concrete is influenced by the porosity of the mix, increase in the porosity of the concrete decreases the strength; further this porosity is mainly attributed by the variation in the w/c ratio in the mix [40]. If the concrete exposed to heating, porosity will be developed in the mortar of the matrix due to the dehydration of the gel phases in the internal morphology. Similarly, in the present case, the concrete developed with natural aggregate and RAC with and without fiber incorporation were subjected to elevated temperature. Concrete specimens (C-N) mix after subjecting to 821 °C, possess a porosity value of 18 %. In the case of concrete developed with RAC possess a porosity value of 20 %. It was seen that 11.5 % higher level was observed for the RAC blended (C-R) mix. As stated above, at this temperature range there will be zero water molecules present in the mix, and further due to the development of pore pressure inside the matrix, tends to increase the porosity percentage. Moreover, the higher porosity level of the RAC blended mix was attributed by the pore formation in the new cement paste as well as the pore formation in the adhered mortar layer in the RAC samples. At 1029 °C, the porosity of the C-N mix was found as 30 %, which is almost 41 % higher than the concrete exposed to 821 °C. Whereas, in the case of RAC blended mix (C-R) mix shows a porosity level of 32 %, which is 37 % higher than the mix blended with natural aggregate (C-N). Fig. 13, shows the porosity range of the concrete specimens with and without fiber based natural aggregate and RAC blended mix.

For the fiber incorporated mixes, C-N-S mix possess a porosity value of 15 % after exposing to 821 °C, which is 17 % lower than the C-N mix; this shows as an evident that, why steel fiber reinforced concrete possesses higher residual strength. For C-N-P and C-N-C mix, the porosity level was 17.34 %, and 14.84 %, respectively. This shows that coconut fiber incorporated specimens possess 14.26 % lower porosity, representing the its superior performance when compared to the polypropylene fiber reinforced mix. Both the C-N-S and C-N-C specimens possess similar behavior when subjected to initial heating for 30 min. In the case of RAC blended fiber reinforced concrete mix, the porosity level was 18 % for C-R-S mix and 19 % for C-R-P mix and 18 % for C-R-C mix. In comparison with its reference specimens i.e. C-R mix; the C-R-S mix shows 9.5 % lower porosity than C-R mix. For C-R-P and C-R-C mix, it shows 5.03 % and 9.26 % lower porosity than the C-R mix. This shows that all the fiber reinforced concrete specimens with RAC outperforms than the reference case in the initial heating of 821 °C. The C-N-S mix shows 18.5 % lower porosity than the C-R-S mix; C-N-P and C-N-C mixes shows 8.63 % and 18 % lower porosity than its counterpart mixes. This shows that aggregate type plays a significant role in the fire behavior of concrete. Further, incorporation of fiber in the mixes clearly shows that integrity of the concrete was still maintained in the RAC based concrete mixes, due to the bridging effect of fibers.

After subjecting to 1029 °C, the C-N-S specimens possess a porosity value of 28 %, which is 6 % lower than the C-N mix. In the case of C-N-P and C-N-C mix, the porosity level was 30 % and 28 %, respectively. When compared to the C-N mix, the C-N-C mix possess 6.4 % lower porosity. Whereas the C-N-P mix resembles the porosity as that of C-N mix. For RAC blended fiber reinforced concrete i.e. C-R-S mix shows 30 % porosity, in addition C-R-P and C-R-C mix possess 31 % and 30 % porosity ranges. C-R-C mix shows 4 % higher porosity than the C-R mix, meanwhile the C-R-P mix resembles the same porosity level as that of C-R mix. In comparison with the natural aggregate blended fiber reinforced mixes, the C-R-S, C-R-P, and C-R-C mix shows 8 %, 4 % and 8 % higher porosity ranges. Increase in the heating temperature increases the porosity range, i.e. C-N-S specimens shows 48 % higher porosity, when heated to 1029 °C, similarly, C-R-S specimen shows 40 % higher loss. 42 % higher porosity was seen for the C-N-P and 39 % for C-R-P, when heated from 821 °C to 1029 °C. Further the rate of increase in porosity level for C-N-C and C-R-C was found to be 47 % and 40 %, respectively.

#### 5.6.6 Porosity correlation

##### 5.6.6.1 Relation between porosity and mass loss

Relation between porosity and mass loss of the concrete subjected to temperature exposure is interlinked each other. Initially, when the concrete subjected to temperature exposure, first reaction taking place inside the concrete is evaporation of free water present inside the concrete pores. Vaporization of free water in the pores/voids in the system tends to reduce the mass of the concrete. After the threshold temperature, the concrete starts to degrade the gel phases, and some phases tends to transform from Ca(OH)_2_ to CaO, which results in porosity development and mass loss [53]. Further, the materials involved for the production of the concrete undergo substantial changes, for instance, concrete developed with carbonate aggregate subjected to higher mass loss when compared to siliceous-based aggregates; carbonate aggregate undergo decomposition of dolomite when subjected to a temperature greater than 600 °C [54]. Decomposition of aggregate inside the concrete further leads to increased mass loss of the concrete and creating pores inside the aggregate. Apart from the aggregate degradation, the internal microstructure of the cement paste tends to decompose at elevated temperature, resulting in pore formation thereby the loss in mass and the strength [55]. Therefore, the pore formation inside the cement matrix has a direct influence on the mass loss as well as strength loss of the concrete.

**Fig. 14:**
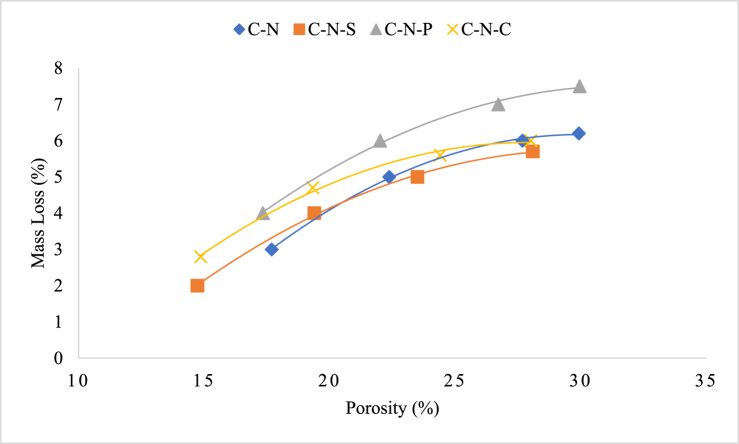
Porosity-mass loss relation of natural aggregate blended FRC concrete subjected to elevated temperature.

**Fig. 15:**
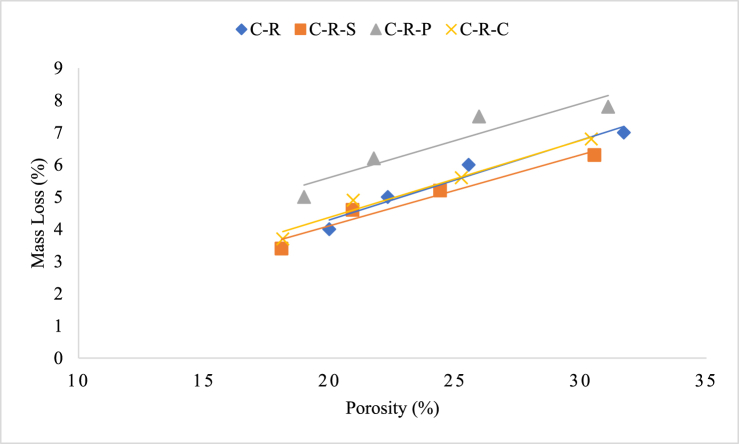
Porosity-mass loss relation of RAC blended FRC concrete subjected to elevated temperature.

In the present study, two types of aggregates were employed for the production of concrete along with the fiber incorporation. From the mass loss-porosity relation, it can be seen that the concrete with polypropylene fiber possess higher range of correlation, representing the concrete was severely damaged when subjected to elevated temperature exposure. Since the exposure temperature was greater than 800 °C, the decomposition rate of the concrete was higher and thus resulting in higher porosity and mass loss. The concrete with polypropylene fiber and coconut fiber incorporation possesses higher rate of degradation; whereas steel fiber reinforced concrete possesses quite lower deterioration when compared to the concrete without fiber. The porosity-mass loss relation of natural aggregate blended FRC was plotted in Fig. 14. The lower degradation of the steel fiber reinforced concrete may be due to high melting point of the steel fiber, when compared to polypropylene and coconut fiber. In comparison with the three types of fiber, steel fiber reinforced concrete possesses higher resistance followed by coconut fiber reinforced specimens. The porosity-mass loss relation of RAC blended FRC after heating was plotted in Fig. 15.

(4) y(C-N-P) = 0.2739× - 0.4489

(5) y (C-N-C) = 0.2377× - 0.3721

(6) y (C-N) = 0.2581× - 1.2508

(7) y (C-N-S) = 0.2743× - 1.7027

(8) y (C-R-P) = 0.2293x + 1.0205

(9) y (C-R) = 0.2473× - 0.6525

(10) y (C-R-C) = 0.2386× - 0.4002

(11) y (C-R-S) = 0.2201× - 0.2934

Equations (4), (5), (6), (7), represents empirical relation of porosity and mass loss of concrete specimens blended with natural aggregate with steel, polypropylene, and coconut fiber. Equations (8), (9), (10), (11), represents empirical relation of porosity and mass loss of concrete specimens blended with RAC with steel, polypropylene, and coconut fiber. The models are based on concrete specimens tested at elevated temperatures between 821 °C and 1029 °C, as per ISO 834 guidelines; as per the guidelines the durations for the corresponding temperatures range from 30 min to 120 min. The models are designed to predict mass loss (%) as a function of porosity (%) in concrete subjected to high temperatures. The values of x (porosity) should be in the range of 10 %–30 % for these models to remain applicable. The exposure temperature must be between 821 °C and 1029 °C with corresponding heating durations of 30–120 min, as specified in the experiment. These models are based on linear relationships between porosity and mass loss. The validity outside of the tested porosity range or beyond the temperature and time conditions may not be reliable.

##### 5.6.6.2 Relation between porosity and strength loss

The porosity of the concrete tends to decrease as the temperature increased from room temperature to 400 °C [56]; during this stage, the hydration reaction of the unreacted cement particles will take place inside the matrix system, which is often called as autoclave curing process. During autoclave curing, the internal microstructure of the concrete becomes compact and denser; which results in strength enhancement of the concrete. When the heating temperature raised to 800 °C, crystallization or dehydration of CSH and other gel phases takes place, due to which the pore structure (micro-pore) converted into macro-porous state by increasing porosity of the concrete matrix [57].

**Fig. 16:**
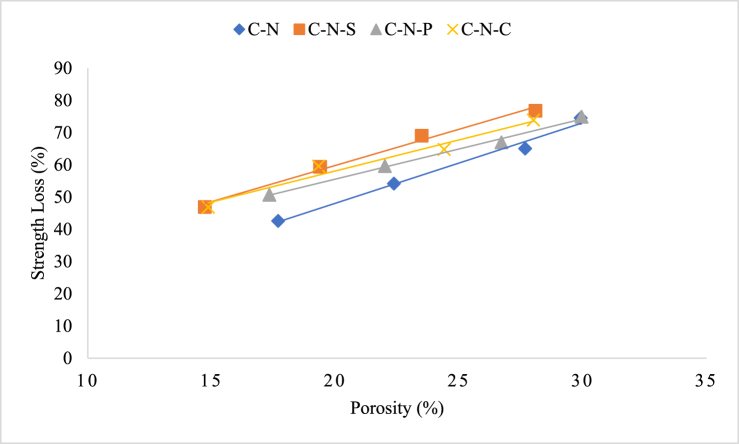
Porosity-strength loss correlation of concrete specimens blended with natural aggregate.

**Fig. 17:**
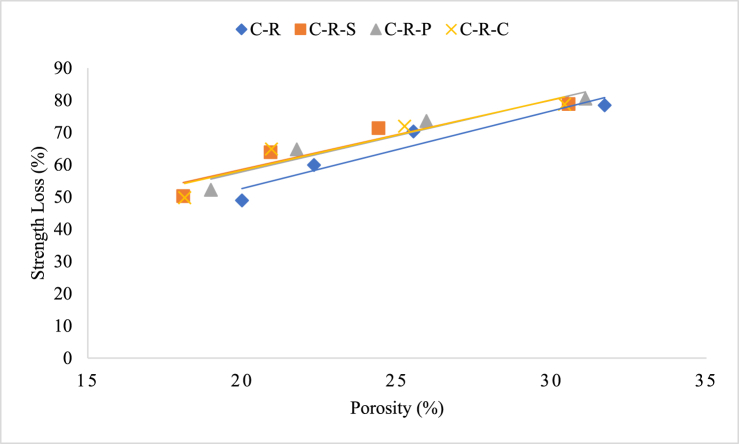
Porosity-strength loss correlation of concrete specimens blended with RAC.

From the experimental investigation, the porosity-strength relation was established for the concrete samples blended with natural and RAC (with and without incorporation of natural and synthetic fibers). From the analysis, it can be seen that C-N-S specimens possess the maximum loss amongst the natural aggregate blended concrete; this could be attributed by the higher initial target strength of the mix, when compared to the mix without fibers. The loss percentage between the C-N-S and C-N mix was found to be 2.8 %, respectively. The porosity-strength loss correlation of concrete specimens blended with natural aggregate was plotted in Fig. 16. Similar scenario was observed for the specimens blended with RAC, comprising natural and synthetic fibers. Strength loss variation of the concrete, as well as the porosity of the concrete was seen to be <5 % for all the mixes, irrespective of the fiber type, for a particular heating intensity. Increasing in the heating intensity tends to alter the loss percentage amongst the mix types (i.e. type of materials employed for the production of concrete) and reinforcing material types. Overall, increasing the percentage of porosity level of the mix was attributed by the intensity and duration of heating the concrete specimens. The porosity-strength loss correlation of concrete specimens blended with RAC after exposing to elevated temperature was plotted in Fig. 17.

Further, it is found that the porosity of the concrete was not only the influencing parameter towards the strength of the mix, when subjected to heating. The strength loss of the concrete was also depending up on the development of pores during the manufacturing process, microcrack (shrinkage cracks) formation during the placement of concrete, and finally, the particle packing mechanism inside the concrete mix. In most cases, porosity is the only factor responsible for the strength loss of the concrete at room temperature as well as under the elevated temperature exposure.

(12) y (C-N-S) = 2.246x + 14.864

(13) y (C-N-P) = 1.8702x + 18.141

(14) y (C-N-C) = 1.9275x + 19.528

(15) y (C-N) = 2.497× - 1.8889

(16) y (C-R-P) = 2.2332x + 13.184

(17) y (C-R-S) = 2.1501x + 15.6

(18) y (C-R-C) = 2.1868x + 14.559

(19) y (C-R) = 2.4071x + 4.5168

Equations (12), (13), (14), (15), represents empirical relation of porosity and strength loss of concrete specimens blended with natural aggregate with steel, polypropylene, and coconut fiber. Equations (16), (17), (18), (19), represents empirical relation of porosity and strength loss of concrete specimens blended with RAC with steel, polypropylene, and coconut fiber. The models are based on concrete specimens tested at elevated temperatures between 821 °C and 1029 °C, as per ISO 834 guidelines; as per the guidelines the durations for the corresponding temperatures range from 30 min to 120 min. The equations proposed above are valid for concrete mixes where the porosity (x) is in the range of approximately 10 %–30 %, based on experimental observation. Further, these models predict the strength loss (y) as a function of porosity within the stated conditions. Moreover, these linear models are empirical and apply specifically to the conditions tested. Extrapolating beyond the temperature of 1029 °C may not yield accurate predictions. And the equations are material-specific to the mixes used (C-N-S, C-R) and may not generalize to other concrete formulations without further validation. Since porosity after fire exposure increases with temperature and time, the boundary conditions ensure applicability within the observed limits of porosity under extreme heat.

#### 5.6.7 Image analysis

Concrete blended with natural and synthetic aggregate (with and without incorporation of fibers) were subjected to 821 °C, 925 °C, 986 °C and 1029 °C, in the present investigation. Subjecting the concrete to a temperature greater than 800 °C, leads to significant impact on the surface, and core. One of the common phenomena in which concrete undergoes after subjecting to elevated temperature is crack development at the surface of the concrete and at the cement paste. Examination of crack in the paste requires nano-scale study; whereas cracks at the concrete surface can be examined in various forms, namely; physical inspection, experimental study through microscopic studies, and through analytical studies. In the present case, open accessed software Image-J was employed to examine the surface modification.

After subjecting the concrete to temperature exposure, the top surface of the concrete was captured and analyzed in the software. Before performing analysis, the RGB based captured image was transformed to grey-scale image [58]. The grey-scale image was processed to form binary image; which is then converted in to threshold image, with respect to Equation (20). Then the image is processed and analyzed to fix the measurement limits, under the area of threshold (limiting value) [59]. After finding the area for the minimum and maximum threshold, surface pores/voids at the surface of the concrete, skeletal mapping were examined by analyzing the particle, to obtain the porosity percentage. The skeletal mapping of the concrete surface was analyzed using Equation (21). From the software analysis, the porosity percentage of the different types of concrete were in the range between 0.88 % and 5.8 %.

(20) $$B_{x,y}=\left\{\begin{array}{c} 0,\gamma_{x,y}<T\\ 1,\gamma_{x,y}\geq T \end{array}\right\}$$

(21) $$s\left(x,y\right)=\left\{\begin{array}{c} 1,\left(x,y\right)\in skeleton\\ 0,Otherwise \end{array}\right\}$$

**Fig. 18:**
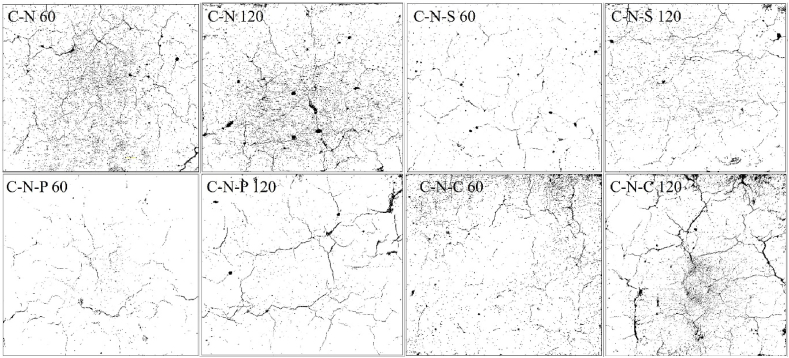
Image analysis of natural aggregate blended concrete after 60 and 120 min of heating.

The C-N mix after subjecting to 60 min of heating shows a surface porosity of 5.06 % and after increasing the heating intensity to 120 min, the surface porosity was seen to be 5.86 %, respectively. The 120 min heated C-N mix possess 13.7 % higher surface porosity than 60 min heated specimens. In the case of C-N-S mix, the surface porosity was found to be 1.2 % after 60 min of heating; whereas for 120 min of heating, the surface porosity was seen to be 2.62 %, representing 53 % higher porosity than 60 min heated case. In the case of C-N-P mix after subjecting to 60 min of heating, the surface porosity was found to be 0.88 %, which is quite lower than all the mixes. Whereas for 120 min of heating, the surface porosity was seen to be 2.24 %, showing 60 % higher than the mix subjected to 60 min. Surface porosity of the C-N-C mix was found to be 3.71 % after subjecting to 30 min of heating; whereas increasing the heating intensity to 120 min possess a porosity value of 6 %, respectively. The 120 min heated specimens possess 38 % higher porosity when compared to 60 min of heating. The image analysis of the natural aggregate blended concrete after subjecting to 60 and 120 min of heating was shown in Fig. 18.

**Fig. 19:**
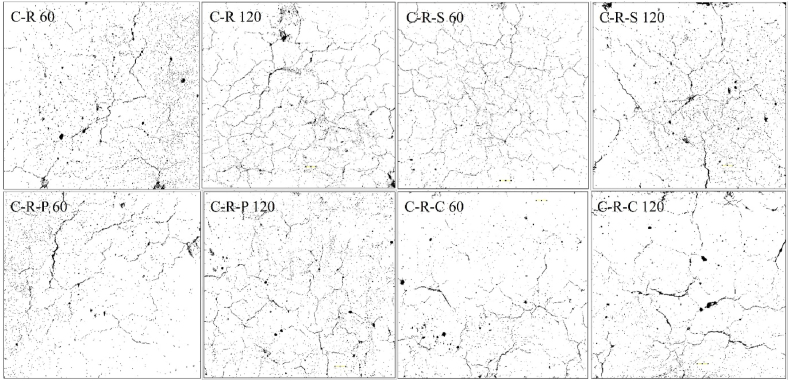
Image analysis of RAC blended concrete after 60 and 120 min of heating.

The porosity of the RAC blended mix i.e. C-R mix exposed to 60 min of heating was 2.35 %, whereas when the concrete subjected to 120 min of heating shows a porosity value of 2.98 %, which is 21 % higher than the mix heated to 60 min. In the case of fiber reinforced RAC blended concrete i.e. C-R-S mix, the porosity after subjecting to 60 and 120 min was 1.95 % and 2.21 %, respectively. The 120 min heated C-R-S mix shows 11.8 % higher porosity level. The C-R-P mix shows a surface porosity of 2.1 % after 60 min of heating and 2.76 % after 120 min of heating; showing 24 % higher porosity level. 1.82 % porosity was obtained for the C-R-C mix after 60 min of heating and 3.12 % for 120 min of heated C-R-C specimens, showing 41.5 % higher than the lower heating duration. The surface analysis of RAC blended concrete after subjected to 60 and 120 min of heating was shown in Fig. 19.

## 6 Discussion and limitation

A. Potential Engineering Applications:

Structural Applications: RAC, reinforced with fibers, can be used in non-critical structural components such as slabs, beams, and columns in low to mid-rise buildings where moderate fire resistance is required. The enhanced fire resistance properties of fiber-reinforced RAC make it suitable for load-bearing members exposed to occasional high temperatures, such as industrial structures, warehouses, and parking garages.

Fire-Prone Infrastructure: The study indicates that RAC, particularly when reinforced with steel fibers, performs well under high-temperature exposure. This makes it viable for fire-prone infrastructure, including tunnels, underground facilities, and structures near wildfire-prone regions.

Sustainable Construction: Given its environmental benefits, RAC can be used in green building projects, where the focus is on reducing the carbon footprint and promoting sustainability. Its ability to maintain acceptable strength and durability after fire exposure also supports its use in eco-friendly construction initiatives.

B. Material Constraints:

Strength Limitations: Despite the benefits, RAC shows reduced mechanical strength compared to natural aggregate concrete (NAC), particularly in mixes without fiber reinforcement. This limitation may restrict its application in high-load bearing structures or critical infrastructure, where maximum strength is a priority.

Porosity and Durability: The study highlights that RAC has higher porosity compared to NAC, particularly after fire exposure. This can affect the material's durability, increasing its susceptibility to environmental degradation such as freeze-thaw cycles, water absorption, and chloride penetration, which may limit its use in structures exposed to harsh weather conditions.

Thermal Degradation: The high strength loss (up to 80 %) after prolonged exposure to elevated temperatures (1029 °C) suggests that RAC may not be suitable for applications where extreme fire resistance is required, such as in high-rise buildings or critical infrastructure like bridges and power plants. The material's limitations under severe fire conditions need to be considered when designing fire-rated structures.

C. Recommendations for Improvement:

Fiber Optimization: While the study shows that steel and coconut fibers improve the fire resistance of RAC, further research is required to optimize fiber content and distribution within the mix. This could help mitigate the high porosity observed after heating and improve both thermal resistance and mechanical strength.

Surface Treatment of Recycled Aggregates: The residual cement paste on recycled aggregates negatively affects the overall strength of RAC. Exploring surface treatments or washing techniques to reduce this residual material could enhance the strength and durability of the concrete.

Hybrid Fiber Composites: Combining different types of fibers (e.g., steel with coconut or polypropylene) in hybrid configurations could offer improved performance by leveraging the complementary strengths of each fiber type. This could provide a better balance between strength, flexibility, and fire resistance.

D. Limitation:

RAC with fiber reinforcement shows significant potential for use in sustainable construction, where fire resistance and environmental considerations are important. However, its limitations, particularly in terms of strength loss and increased porosity after fire exposure, should be addressed through material optimization and more controlled applications in non-critical structural components.

## 7 Conclusion

1. Workability and Density: The use of RAC led to a decrease in workability compared to natural aggregate concrete, and further incorporation of fibers reduced workability. However, the density remained largely unaffected, with only a minor reduction in RAC-based mixes, indicating no significant impact on concrete's overall density.
2. Durability Properties: Both RAC and natural aggregate concrete demonstrated marginal variations in sorptivity, with less than a 5 % fluctuation. The porosity of RAC was slightly higher (2 ± 0.5 %) compared to natural aggregate concrete, yet this difference was within acceptable limits for structural applications.
3. Mechanical Performance: RAC, when combined with steel fibers, achieved target compressive strength similar to natural aggregate concrete. Coconut fibers exhibited strength behavior comparable to polypropylene fibers, suggesting that coconut fibers could serve as a sustainable alternative in structural concrete.
4. Thermal Resistance: After exposure to elevated temperatures, both RAC and natural aggregate concrete showed significant strength loss, with up to 50 % loss after 30 min of heating, increasing to 74–80 % after 120 min. Crack widths and porosity levels were higher in RAC mixes post-heating, with maximum porosity reaching 32 %, yet the material maintained reasonable performance under thermal stress.

Scope for future studies:

• Impact of multiple cycles of heating and cooling (thermal fatigue) to simulate real-world conditions
• Peak stress, peak strain, ultimate strain, elastic modulus, and constitutive material behavior for recycled aggregate concrete blended with and without fibres.
• Further research could investigate the use of other natural fibers, such as bamboo, jute, or sisal, for potential use in concrete
• Investigating the synergistic effect of combining two or more types of fibers (both synthetic and natural)
• Investigation into the changes in microstructure (e.g., pore size distribution, micro-cracking) of concrete exposed to high temperatures using techniques like scanning electron microscopy (SEM)

## CRediT authorship contribution statement

**Balamurali Kanagaraj:** Writing – review & editing, Writing – original draft, Investigation. **Shinu Shaji:** Investigation, Data curation. **Meshach Jafrin:** Investigation, Data curation. **Samuvel Raj R:** Investigation, Data curation. **N. Anand:** Writing – review & editing, Supervision, Methodology, Conceptualization. **Eva Lubloy:** Investigation, Funding acquisition, Formal analysis.

## Data and code availability statement

Data included in article/supplementary material is referenced in the article.

## Declaration of competing interest

The authors declare that they have no known competing financial interests or personal relationships that could have appeared to influence the work reported in this paper.
